# Rapid and sensitive detection of *Yersinia pestis* by lateral-flow assay in simulated clinical samples

**DOI:** 10.1186/s12879-018-3315-2

**Published:** 2018-08-14

**Authors:** Hui-Ling Hsu, Chuan-Chang Chuang, Chung-Chih Liang, Der-Jiang Chiao, Hsueh-Ling Wu, Yu-Ping Wu, Feng-Ping Lin, Rong-Hwa Shyu

**Affiliations:** Institute of Preventive Medicine, National Defense Medical Center, P.O. Box 90048-700, Taipei, Taiwan

**Keywords:** Bronchoalveolar lavage, Capsule-like F1 antigen, Immunogold detection, Lateral flow assay, *Yersinia pestis*

## Abstract

**Background:**

*Yersinia pestis* is a contributing agent to the epidemic disease, plague, which killed an estimated 200 million people during historical times. In this study, a rapid, cheap, sensitive, and specific technique, the lateral flow assay (F1 strips), has been successfully developed to detect this pathogen, by using paired monoclonal antibodies (MAbs) against *Y. pestis* capsule like fraction 1 (F1) protein. Compared with the polyclonal antibody (PAb) based F1 strips, the Mab-based F1 strips have a remarkable increased detection limitation (10 to 100 folds). Furthermore, besides the limitation and specificity evaluation, the application of this F1 strip on simulated clinical samples indicate the LFA can be a good candidate to detect plague.

**Methods:**

Recombinant F1 antigen was expressed and purified from a series of works. The various anti-F1 monoclonal antibodies generated from hybridoma cells were screened with the ELISA technique. To evaluate the feasibility of this *Y. pestis* F1 test strip, the F1 protein/*Y. pestis* was spiked into simulated clinical samples such as human serum, mouse bronchoalveolar lavage fluids, and mouse blood to mimic natural infection status. Additionally, this technique was applied to detect the *Y. pestis* in the environment-captured rats, to evaluate the practical usefulness of the strips.

**Results:**

By using this MAb-based-LFA technique, 4 ng/ml of recombinant F1-protein and 10^3^ CFU/ml of *Y. pestis* could be detected in less than 10 mins, which is at least 10-folds than that of the PAb format. On the other hand, although various *Yersinia* strains were applied to the strips, only *Y. pestis* strain showed a positive result; all other *Yersinia* species did not produce a positive signal, indicating the high efficiency and specificity of the MAb-based F1-strips.

**Conclusion:**

Based on our findings, we suggest that the MAb-format-LFA will be valuable as a diagnostic tool for the detection of *Y. pestis*. This report shows that the F1 strip is sufficient to support not only the detection of plague in simulated clinical samples, but also it may be a good candidate to meet the epidemiological surveillance during an outbreak of the biological warfare.

## Background

*Yersinia pestis* is a slow-growing, non-motile, and non-spore-forming gram-negative coccobacillus (0.5 ~ 0.8 μm in diameter) of the family Enterobacteriaceae. It is regarded as a facultative extracellular pathogen and is the causative agent of the notorious plague [1]. Plague (the Black Death epidemic caused by *Y. pestis*, 1347–1351), is a known flea-borne disease that can trigger large epizootics among the rodent population. Humans that live in environments close to these rodents can contract the bubonic plague either through direct contact with infected animals or through transmission by infective flea bites [2, 3]. Although the bubonic and septicemic plague is not proven to be epidemical, it is possible for patients to transmit the pneumonic plague. This is the most serious form that can produce highly contagious aerosols in the environment; the distance required for effective transmission is only 2-m [4, 5]. In addition, if antibiotic treatment is not administered within 24-h after the onset of symptoms [6, 7], the pneumonic plague can become lethal, caused more than 200 million total deaths worldwide [4, 8, 9]. Over the last decade, thousands of cases of plague have been reported to the WHO (World Health Organization) annually, indicating that the plague has not been eliminated. This is especially true in places where public health and living conditions are poor [10]. Owing to the high virulence and the high mortality rate due to *Y. pestis* infections, this species has the potential to be developed as a biological weapon [1, 11]. Hence, it is currently categorized as a restricted agent (A-list) by the CDC (Centers for Disease Control and Prevention, Atlanta, USA). Plague might cause outbreaks and missed by clinical diagnosis, hence it is of great significance to establish a convenient approach for the detection of *Y. pestis* (or the antibody that against *Y. pestis* F1-antigen) from infected/suspected cases. This study would meet the requirement of epidemiological surveillance on site and/or during biological warfare, and could reduce mortality, through early monitoring and adequate treatment, and effectively control distribution of the infectious agent.

*Y. pestis* can secrete a capsule-like surface antigen called the fraction 1 protein (F1 antigen) after infection [12]. The F1 antigen was demonstrated to be a qualified marker for identification of *Y. pestis* [13], and it contributes to phagocytosis resistance in *Y. pestis* [5]. Antibodies generated against F1 seem to be closely associated with protection from plague [14, 15]. In most cases, plague is transmitted within the rodent population without significant disease signs, yet antibodies to F1 are always detected [15, 16]. Previously, studies have reported that *Y. pestis* can be confirmed simply by detecting antibodies against the F1 antigen [17, 18]; Chanteau et al. has also showed that levels of the F1 antigen, present in the sera of plague patients, can range from as low as 4 ng/ml to 50 μg/ml [18]. Therefore, it is appropriate to develop a highly sensitive and specific assay for F1 detection, both for epidemiologic surveys of the plague and for screening *Y. pestis* transmission within natural animal reservoirs.

Several well established assays have been applied to detect *Y. pestis*/F1 protein, such as the indirect hemagglutination assay (IHA, which is the current gold standard for *Y. pestis* detection) [19], enzyme- linked immunosorbent assays (ELISAs) [20], polymerase chain reaction (PCR) analysis [21], and fiber optic biosensor measurement [22, 23]. Although most reports have focused on identifying the bacterial species, these techniques are all highly sensitive and specific. Nevertheless, costly equipment (such as ELISA readers, PCR machines, and computers, among others), relatively long assay times (no less than several hours), the need for specialized technicians, and the complex dynamic analysis procedures limit the performance of these assays for detection of contagious plague foci. To minimize these deficiencies and to mitigate the restrictions of analysis, a low-cost, rapid, sensitive, and accessible immunoassay for the detection of low levels of *Y. pestis* in habitats of humans and rodents is urgently required. The lateral flow assay (LFA) appears to have the potential to fulfill these requirements.

The LFA, also known as the immunochromatographic assay or the strip assay, has been verified for use in numerous analytical tests such as those for hormones [24] and allergens [25], drugs [26], antibodies [27], and toxins [28, 29]. This assay is an immunochromatographic process based on the immunological reaction of the double-antibody sandwich format on a porous nitrocellulose (NC) membrane [29–31]. Among several immunological assays (including LFA, ELISA, flow cytometry, immunofluorescence microscopy, etc.), LFA was proved to be the best identification technique for on-site testing by untrained personnel [17]. Besides, LFA also has several benefits, such as its easy-to-use format, very short assay time (generally less than 10-min), long-term stability over a wide range of temperature, and relatively low production expenses. In addition, this technique does not require special training, skilled technicians, specific equipment, or animals. Furthermore, LFA results can be visualized directly by the naked eye. These characteristics imply that the LFA is an appropriate technique for on-site testing by untrained personnel.

Bronchoalveolar lavage (BAL) has been widely used to sample the contents of the epithelial lining fluid. For example, Drent et al. [32, 33] reported the use of BAL in the diagnosis and management of sarcoidosis, and Nagai et al. [34] discussed the use of BAL in evaluating idiopathic interstitial lung diseases. On the other hand, blood is the most convenient and commonly assayed body fluid. Hence in the present study, we utilized the MAb format F1-strip to detect *Y. pestis* in these simulated clinical samples (from experimental mice and wild-caught rats). The results demonstrated that MAb format F1-strip is an ideal candidate for detection of *Y. pestis* in the environment and clinic.

## Methods

### Construction of recombinant p18RMAF1

Two DNA fragments containing the *Y. pestis caf* operon [35, 36], were amplified by PCR (Perkin Elmer DNA Thermal cycler 480; 30 amplification cycles: 95 °C for 1-min, 54 °C for 1-min, and 72 °C for 2-min) with *Y. pestis* ATCC 19428 template DNA and oligonucleotide primer pairs including: F1OU(F) (5′-GTTCCGGAATTCTTCCGAACATAAATCGGTTCAGTGGCC-3′) and F1OU(R) (5′-GGCGTA TTCCTCGCTAGCAATGTTTAACG-3′), or F1OD(F) (5′-ATCGTTAAACATTGCTAGCGAGGA ATACGCC-3′) and F1OD(R) (5′-GTTCCGCTGCAGTGAACCTATTATATTGCTTCGCGC-3′). Amplified DNA fragments were first ligated together using the unique *Nhe* I restriction site in the *caf* operon, and then cloned into a high-copy-number vector, pUC18 that obtained from Promega (Medison, WI, USA). This was followed by *Eco* RI/*Pst* I digestion (with T4 ligase, Invitrogen) to generate the plasmid *p*18RMAF1. To confirm the coding sequences of *caf* operon genes, DNA sequencing was performed, and the deduced amino acid sequences of the *caf* operon genes of *Y. pestis (*ATCC 19428) were shown to be identical to previously reported sequences of *Y. pestis* CO92 (Accession No. AL590842, data not shown). The recombinant *p*18RMAF1 plasmid was transformed into *E. coli* strain DH5 to generate the *E. coli* (*p*18RMAF1) strain.

### Expression and purification of recombinant F1 antigen

All cultures, tests, and treatments involving *Y. pestis* were performed in a bio-safety level 3 (BSL-3) laboratory. For expression of the F1 antigen, a seed culture was prepared as follows (culture medium (Luria-Bertani broth, LB) was purchased from BD (Becton Dickinson, USA)): LB broth (10 ml) containing ampicillin (100 μg/ml) was inoculated with a loop of *E. coli* (p18RMAF1) from a plate stock, and grown for 16~ 18-h at both 28 and 37 °C with shaking. LB broth (2-L) containing ampicillin was subsequently inoculated with the seed culture and grown at 28/37 °C for 48-h with shaking at 200 rpm. The cultures were harvested by centrifugation at 12,000×*g* for 5-min at 4 °C. Following centrifugation, ammonium sulfate (final concentration 30% w/v) was added to the bacteria-free supernatant to generate the F1 precipitant (4 °C, overnight). The F1-containing precipitate (if there contains any) was harvested by further centrifugation (12,000×*g*), dissolved and dialyzed (overnight at 4 °C) against 1 mM PBS (potassium buffer saline, pH 7.2), followed by filtration through a 0.22 μm membrane. Finally, the crude F1 antigen was further purified through a low-molecular-weight cutoff filter (10 kDa, Amicon Ultra, Millipore Co. Bedford, MA), a size-exclusion chromatography Superose-6 column (SEC, separation based on molecular size, GE Healthcare), and an anion-exchange Q-Sepharose FF column (AEC, separation by molecular charge, GE Healthcare) [37]. The purity of the F1 protein was evaluated using sodium-dodecyl-sulfate-polyacrylamide gel electrophoresis (SDS-PAGE, 12%) and western blotting [38], using a mouse anti-*Y. pestis* F1 antibody (YPF19, Abcam Inc). Finally, the F1-protein was quantified by Coomassie Plus – The Better Bradford Assay Kit (Thermo Scientific, USA).

### Generation of anti-F1 monoclonal antibodies

Monoclonal antibodies against the F1-antigen were generated as previously described [39]. Briefly, F1-antigen (100μg/ml, 200 μl) was first mixed with an equal volume of complete (first inoculation) or incomplete (subsequent inoculation) Freund’s adjuvant (Difco). The antigen-adjuvant mixture was then subcutaneously injected into BALB/c mice. The eight-week-old BALB/c mice (male, number of mice: 5) for ascites production were purchased from Biolasco, Taipei, Taiwan. After 4 weeks, the mice were boosted with F1-antigen (0.1 mg/ml, 100 μl) by intravenous inoculation. Mice were sacrificed (inhalation with isoflurane and then CO_2_ suffocation) 3 days after the intravenous boost, and spleens were removed and homogenized. The spleen cells were subsequently fused with mouse myeloma NS-1 cells to produce hybridoma cells.

For hybridoma selection, culture medium collected from hybridoma clones was centrifuged at 500×*g* for 10-min, and the supernatants were assayed by the ELISA screening. First, flat-bottom 96-well plates (ICN Biomedicals) were coated with 50 μl of purified F1 protein (2 μg/ml) in 0.05 M NaHCO_3_ (pH 9.6), at 4 °C overnight. The plates were decanted, washed (dd H_2_O) and blocked with 200 μl of blocking buffer (5% BSA in PBST (PBS containing 0.1% Tween-20)) at 37 °C for 1-h. Subsequently, the wells were washed four times with 250 μl of PBST, and were incubated with 50 μl of culture supernatant for 1-h at 37 °C. The plates were washed again (4×) with 250 μl of PBST, and incubated with 50 μl of goat-anti-mouse IgG (H + L)-HRP at a 1:2000 dilution in PBST for 30-min at 37 °C, followed by decanting and final washing. Substrate, 50 μl sodium perborate (Sigma P-4922) in phosphate-citrate buffer containing O-phenylenediamine (Sigma P-6787) and 0.03% H_2_O_2_, was added. After 10~ 20 min incubation in the dark (room temperature), the reaction was stopped by adding 100 μl 4 N H_2_SO_4_ and the absorbance was determined by ELISA reader at 490 nm.

After selection, the hybridoma cells were injected intraperitoneally into five BALB/c mice with incomplete adjuvant for ascites production. Various anti-F1 monoclonal antibodies were purified from mice ascites through an IgG-specific immunosorbent, thiophilic gel (Pierce, Rockford, USA). (5).

### Animal operations

The animals (for ascites production) were placed as a whole (i.e. housed in a 362 × 235 × 195 mm cage with sufficient food and water) and given 5 days to accommodate the environment in the housing facility. The environmental conditions were as followed: temperature: 21 °C. ± 2 °C., humidity 55% ± 10%, illuminance 350 lx, ratio of light to dark cycle was 1:1, and light was turned on and off at 0700 and 1900 h. All animal experiments were conducted in compliance with the regulations of IPM Institutional Animal Care and Use Committee (IPM-IACUC) of the National Institute of Allergy and Infectious Diseases, National Institutes of Health, and with licenses of IPM-IACUC ref. AN102–15 (2013) and AN-104-15 (2015). All sections of this report adhere to the ARRIVE Guidelines for reporting animal research.

A completed ARRIVE guidelines checklist is included as an addition file.

When ascites has been taken twice, or when the tumor was too big to affect physiological conditions (e.g., activity, breathing, etc.), the experiment is terminated and euthanized by anesthesia (with isoflurane) to prevent animals from to suffering. The animals are then packaged in plastic bags, autoclaved and subsequently incinerated.

### Conjugation of monoclonal anti-F1 antibody to colloidal gold particles

Monoclonal anti-F1 antibodies were conjugated to colloidal gold particles (acquired from Aurion (Wageningen, Netherlands)) as described previously [29, 31, 39]. Briefly, 25 nm colloidal gold particles were applied to the conjugation process. The anti-F1 IgG (1 mg/ml, 0.1 ml) was gently added to 0.9 ml of colloidal gold solution (1% w/v, pH 8.5) and incubated with shaking for 30-min at room temperature. The colloidal gold particles were next precipitated by centrifugation for 30-min at 4 °C (1550×*g*; 8178 swing-out rotor, Labofuge 400R, Heraus Instrument, USA), and suspended in 1 ml working buffer (20 mM Tris/HCI buffer (pH 8.2) containing 1% w/v BSA). The optical density of the suspension was adjusted to 5.0 at 520 nm. The prepared anti-F1 IgG-coated colloidal gold probes were stored at 4 °C until use (30 μl/cm on the conjugation pad).

### Preparation of immunochromatographic test strips

The strip elements, including high-flow nitrocellulose membranes (AE-98), glass fiber conjugation pads (AccuFlow™G), sample application pads (#12-S), and reagent adsorption pads (470 Zuschnitte), were all purchased from Schleicher & Schuell GmbH (Dassel, Germany) and had been described previously [28, 29, 31]. Briefly, 1 μl of goat anti-mouse IgG (whole molecule, purchased from Sigma (St. Louis, MO, USA)) and mouse anti-F1 monoclonal antibodies (1 mg/ml each) were sprayed onto a nitrocellulose membrane independently using a BioDot dispensing apparatus (BioDot XYZ 3000 1414) to create a control region (C) and a test region (T). The membrane was incubated in 1% w/v polyvinyl alcohol for 30-min at room temperature to block remaining active sites, followed by a quick wash of the strip with ddH_2_O and subsequent drying. The membrane was then adhered to an adhesive paper plate (2.44 × 11.81 in., Adhesive Research Inc., Taiwan), with an additional reagent adsorbent pad, a colloidal gold conjugate pad (containing F1-IgG probes), and a sample application pad. The paper plate was then cut into 5-mm-wide strips (CM4000 cutter, BioDot) and mounted on a plastic cassette. At this point, the device was ready for use.

### Bronchoalveolar lavage fluid collection

The extraction of mouse BAL fluid was based on the method described by Baughman [40], with a minor modification. Briefly, two 12-week-old ICR mice (male, Biolasco, Taiwan) were anaesthetized with 0.03 ml Zoletil-50 (Zolazepam + Tiletamine: 2.5 × dilution with PBS, pH 7.4) through intramuscular injection, followed by heart exsanguination (blood was aliquot in two tubes containing anticoagulant EDTA). The mice were then anatomized, cut the neck skin and pushed back the neck muscles to expose the trachea. An IV catheter (24 GA, 0.75 in, Angiocath Plus™, BD REF 591836, Korea) was then inserted, fixed with the black nylon threads, washed twice with normal saline (1 and 0.5 ml each), and the lavage fluids (1.2 ml final volume) were collected.

The living environment for ICR mice was identical to that of the BALB/c mice.

### Sensitivity and specificity of the F1 test strips

Sensitivity and specificity assays for the test strip have been reported elsewhere [29, 31, 41].

Briefly, appropriate amounts (100 μl) of various concentrations of F1 proteins (2~ 20 ng/ml), or *Y. pestis* (10^3^~ 10^5^ CFU/ml as measured by plate count) were applied individually to the strips. To facilitate sample migration, a half volume (50 μl) of tracing buffer (potassium carbonate buffer, PCB) was applied when samples were almost drained. In addition, various samples containing *Yersinia* strains (e.g. *Y. pestis yreka* strain IPM00722; *Y. mollaretii* (ATCC43969); *Y. frederiksenii* (ATCC 29912); *Y. pseudotuberculosis* (ATCC29910); *Y. enterocolitica* (ATCC 27729) and *Y. intermedia* (ATCC29909) were also assayed by F1 strips for specificity evaluation. The Enterobacteriaceae bacterial strains were kindly supplied by Dr. Shih-Shiung Huang, from the Institute of Preventive Medicine, National Defense Medical Center, Taiwan. To verify the reproducibility of the strip analysis in this section, the sensitivity and specificity tests were performed in triplicate from the same (or different) batch of strips (intra- and inter-assay).

### Evaluation of the F1 test strip with simulated and field-captured rat samples

To assess the feasibility of F1-strips in various biological conditions, several different matrices were employed. The samples (100 μl of F1 or *Y. pestis*) were mixed with the appropriate volume of human sera (purchased from Sigma: S1-M EMD Millipore, Sigma-Aldrich, 1:5 dilutions in PCB), mouse blood (0.5 ml from one BALB/c mouse, 1:10 dilutions in normal saline), or mouse BAL (1:10 in normal saline). Mouse blood was pre-treated with distilled water for 10 min to allow the blood cells to burst and hence reduce interference and false-positive test results, and subsequently diluted with normal saline. Control samples were prepared by direct application of PCB/normal saline to the matrices. The F1-strips were also evaluated using sera from the rodents captured in Penghu Island (48 rodents, 13 with flea). The rodents were anesthetized before exsanguination, and same as before, the sera were diluted with five volumes of PCB followed by applying onto the homemade Mab/Pab F1-strips. In order to validate the efficiency of the F1-strips, samples were also analyzed by ELISA. The F1 immunized and pre-immunized NC-C2 rats were used as the control group.

## Results

### Construction, expression, and purification of recombinant F1 protein

**Fig. 1:**
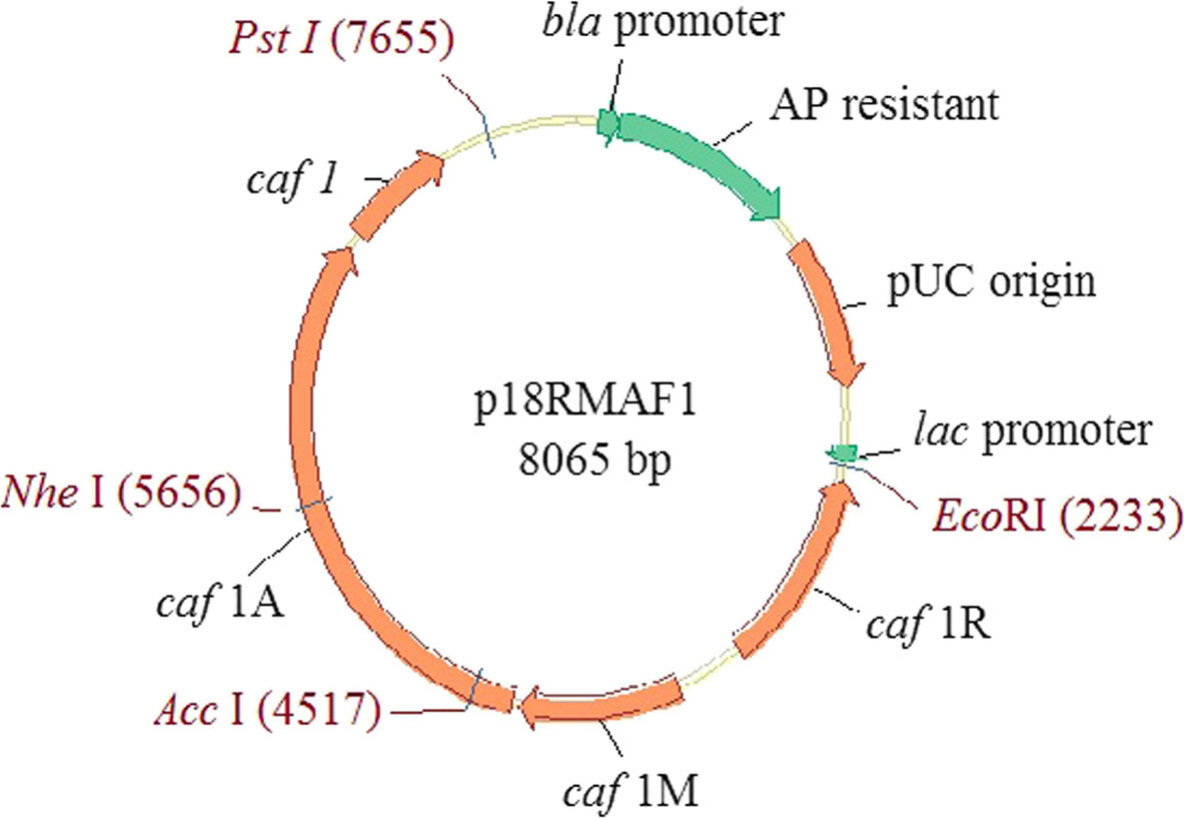
The structure of *Caf*1 recombinant protein expression vector p18RMAF1. The *caf* operon from *Y. pestis* encoding the structural subunit (*caf*1), the molecular chaperone (*caf*1M), the outer membrane anchor (*caf*1A) and the regulatory protein (*caf*1R) were cloned into the vector pUC18 to form p18RMAF1. (Abbreviations: Ap - ampicillin, lac - lactose)

Figure 1 shows the constructed plasmid. Three structural subunits, *caf1*, *caf1M*, *caf1A*, and the regulatory protein (*caf1R*) were cloned into the pUC18 vector to generate the 8-Kb recombinant plasmid p18RMAF1. The *caf* gene cluster could thus express the F1 structural protein. This plasmid was then transformed into *E. coli* for F1 protein production.

The F1 protein was subsequently expressed and purified as described in the materials and methods section. To test the presence of F1, we cultivated the *Y. pestis* at different temperatures (37 °C and 28 °C, same incubation time). A previous study mentioned that “F1 capsule production is strongly influenced by temperature--little or no capsule is detected at < 35°C in vitro or in the flea vector” [42]. The cultivation results show that F1 can only be detected in the bacterial cells cultured at 37 °C, but not at 28 °C (similar bacteria concentration, judged by McFarland detection kit), which was consistent with the previous report (data not shown).

**Fig. 2:**
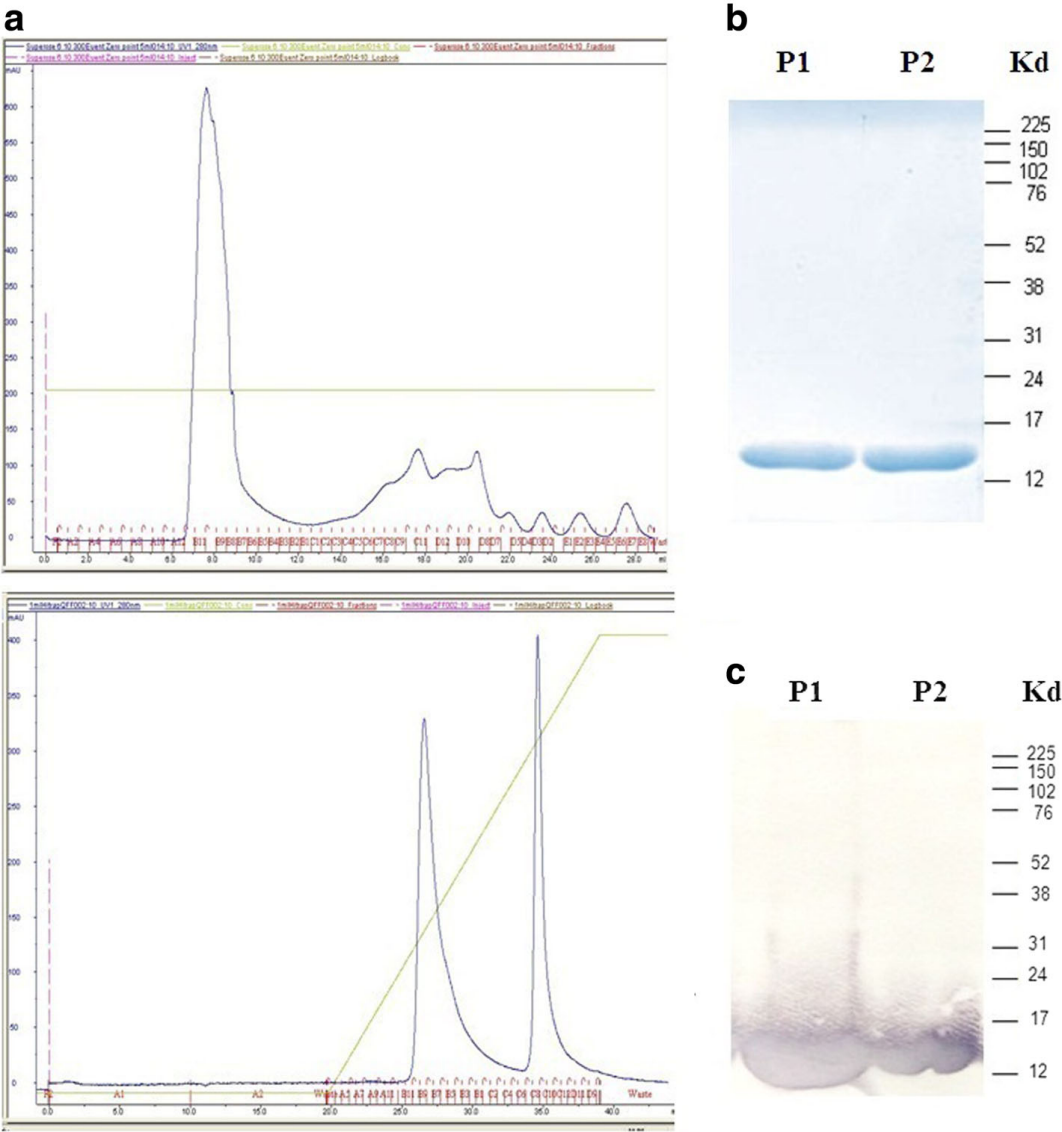
**a**. Superose 6 column(upper) and Q-Sepharose column (lower) purified F1 protein. P1 and P2 fractions were subsequently pooled together as immunized antigen. **b**: SDS-PAGE of *E. coli*-derived recombinant F1 protein purified from Q-Sepharose column. **c**: Western blot assay of *E. coli*-derived recombinant F1 probed with anti-F1 mouse serum. 1st Ab: Anti-F1 Mab (YPF19); 2nd Ab: AP-conjugated anti-mouse IgG

After Superose (for size exclusion, one peak) and Sepharose (for ion exchange, two peaks) purification (Fig. 2a), the expressed F1 recombinant protein was assayed by SDS-PAGE followed by western blotting. If the order of these two columns was changed, no difference in purity was observed (data not shown). The results of SDS-PAGE (Fig. 2b) and western blot (Fig. 2c) showed that P1 and P2 had the same pattern and both displayed the predicted molecular weight of F1-antigen (15.5 kDa) [11]. P1 and P2 were then pooled and used as an antigen for mice immunization.

### Production of F1-MAbs

For F1-Mabs production, the ELISA screening was performed for F1-specific hybridoma clone selection. Hundreds of anti-F1 hybridoma clones were screened in this assay, and only five of which were selected (OD_490_ > 1) and were shown in Table 1. After selection, the hybridoma cells were injected into BALB/c mice for ascites production. These high-titer Mabs had the potential (but not fully qualified) to meet the requirements for the strip assay. All selected Mabs were then paired (either by capture or detection form) on the assay strip to obtain the most suitable combination.

**Table 1:** The spectral absorption values for five selected anti-F1 monoclonal antibodies. Four of which have OD_490_ value over 1.0 and the rest one was a little below. All these MAbs were applied onto the strip assays for the optimum pairing

Monoclonal antibodies	Absorbance of OD_490_
F1-4B5–3	1.219
F1-5D5–2	1.209
F1-5F3–2	1.298
F1-6B3–2	1.321
F1-9D1–2	0.797

### Pairing of MAbs on strip device

The five selected monoclonal antibodies were conjugated to colloidal gold particles individually as the capture antibody, and each of them was also sprayed on the NC-membrane as the detection antibody. Every capture-detection combination on the strip device formed the sandwich format, which was then used for sensitivity evaluation of the strips. A comparison of each antibody as the capture or detection antibody yielded a small difference (Table 2). In this assay, two antibodies, 4B5–3 and 5F3–2, were identified as the optimal permutation, and were applied to strips for subsequent analysis. The results of this pairing evaluation are presented in Table 2.

**Table 2:** Pairing of monoclonal antibodies to evaluate the most appropriate combination. The results revealed that the most suitable composition was 4B5–3(capture) versus 5F3–2 (detection), which would apply for the subsequent strip assays

		Detection antibody				
		F1-4B5–3	F1-5D5–2	F1-5F3–2	F1-6B3–2	F1-9D1–2
Capture antibody	F1-4B5–3		+	**+++**	+	–
	F1-5D5–2	–		+	–	–
	F1-5F3–2	++	+		+	–
	F1-6B3–2	+	–	+		–
	F1-9D1–2	–	–	+−	–	

### Sensitivity and specificity of F1 test strips

**Fig. 3:**
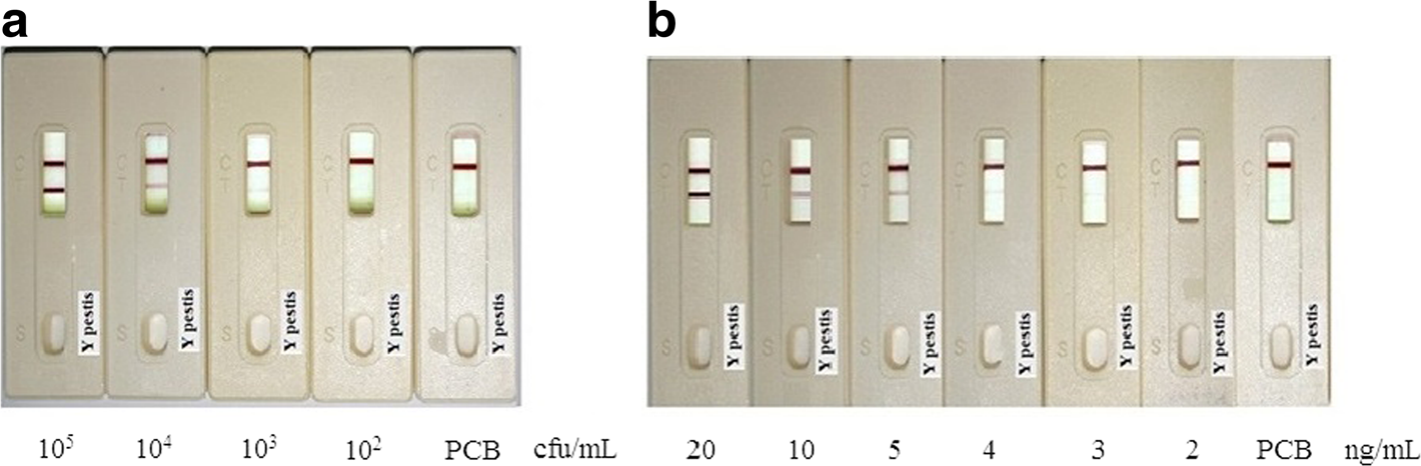
Sensitivity assay of *Y. pestis* F1-strips. Series dilutions of (**a**) *Y. pestis* and (**b**) F1-protein in PCB were applied on the *Yersinia* F1-strips. The strips can successfully detect *Y. pestis* and F1 with detection limit of 10^3^ CFU/ml and 4 ng/ml, respectively

**Fig. 4:**
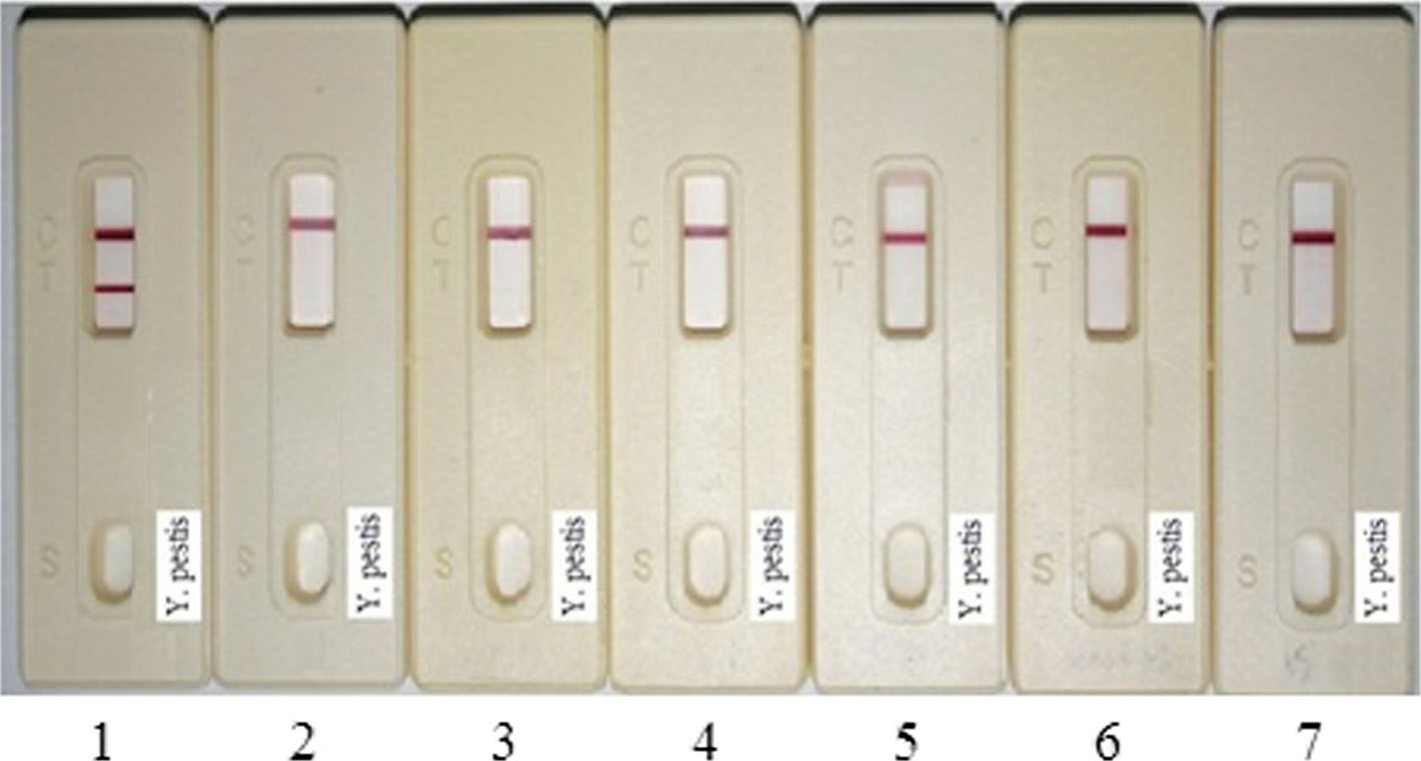
Specificity assay of *Yersinia* F1-strips. Six *Yersiaia* strains (10^5^ CFU/ml each) were applied on *Yersinia*-F1-strips. Strip 1. *Y. pestis yreka* strain IPM00722; 2. *Y. mollaretii* (ATCC43969); 3. *Y. frederiksenii* (ATCC29912); 4. *Y. pseudotuberculosis* (ATCC29910); 5. *Y. enterocolitica* (ATCC27729); 6. *Y. intermedia* (ATCC29909); 7. PCB buffer

For the sensitivity assay of F1 test strips, samples with different amounts of *Y. pestis* and F1 protein were applied to previously prepared F1-strips. Figure 3 presents the assay results. All the analyses were accomplished in less than 10-min, and the detection limit for the bacteria and F1 protein was 10^3^ CFU/ml and 4 ng/ml, respectively. While in the absence of *Y. pestis*, there was no visible band in the test area and was therefore considered as negative. For specificity analysis, various *Yersinia* strains (10^5^ CFU/ml of each) were applied to the F1-strips. Figure 4 shows that only *Y. pestis* presented a positive result, whereas all other *Yersinia* species resulted in negative. As for inter- and intra-assays, at least three assays were included. This means that the intra-assays were to test the strips generated from the same batch at different times (e.g. once a week, at least 3 weeks). The number of strips used per batch was not less than 30. As for inter-assays, strips produced from different batches (at least 3 different batches, under the same generation conditions) were assayed simultaneously. Likewise, at least 30 strips were used for each assay. All tests results were confirmed by the six members of the experimental group. In addition, in this section, no sensitivity or specificity variation occurred in the inter- and intra-assays.

The sensitivity and specificity assay results demonstrated that this format has a remarkable increased detection sensitivity compared to that of Pab-based methods [39]. That is, in the previous study, a LFA based on a polyclone-antibody format for detection of *Y. pestis* F1 antigen and bacteria obtained the detection limit of 50 ng/ml and 10^5^ CFU/ml, respectively. Whereas in this study (MAb-based), the detection sensitivity was improved to 4~ 5 ng/ml for F1 antigen and 10^3^ CFU/ml for bacteria. Although silver enhancement may improve the detection sensitivity; it needs at least 1 h to obtain results.

If there is sufficient time, for sure it can be applied in field situations thus increase the detection limit; or it needs to be proceeded on a laboratory bench in other ways.

### Evaluation of simulated sample and wild-captured rat serum by F1 test strips

**Fig. 5:**
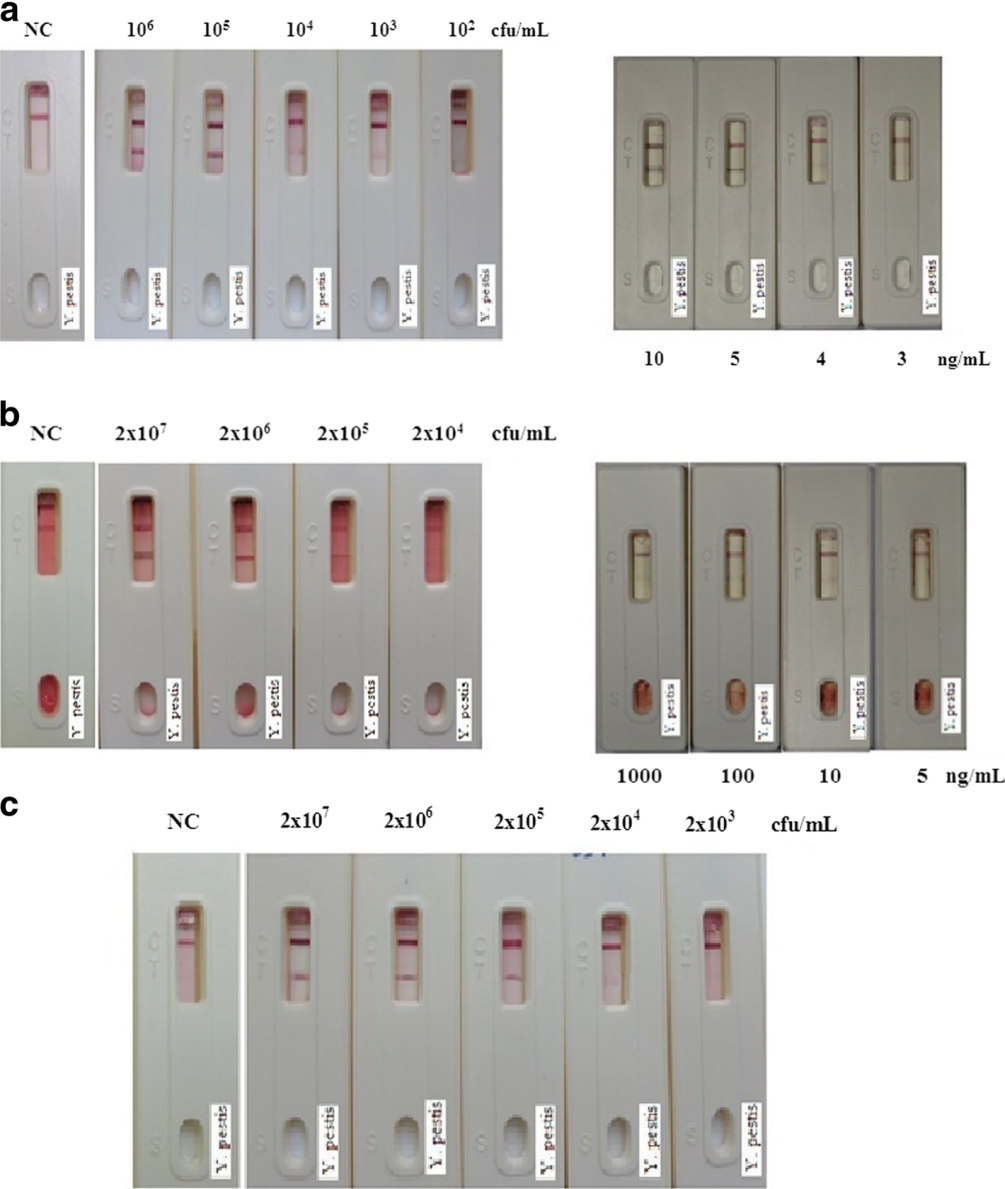
Sensitivity assess of F1/*Y. pestis* in various matrices. F1 protein/*Y. pestis* spiked into three simulated samples were assayed by *Yersinia* F1-strips. All the samples were diluted in the control reagents such as (**a**). human serum: 1:5 dilutions in PCB; Left: *Y. p.* test; Right: F1 test. (**b**). mouse blood: 1:10 dilutions in normal saline; Left: *Y. p.* test; Right: F1 test. (**c**). mouse BAL: 1:10 dilutions in normal saline. The human serum had the same detection limit (10^3^ CFU/ml and 4 ng/ml) as that in PCB; while for mouse blood and BAL, the detection sensitivity has decreases to some extent. (NC: normal saline control)

F1 protein or *Y. pestis* spiked into human serum, mouse blood, and mouse bronchoalveolar lavage fluid was assayed by the F1-strips. Results (Fig. 5 and Table 3) revealed that in human serum, both F1 protein and *Y. pestis* maintained the same detection sensitivity as that in PCB (Fig. 5a); however, some sensitivity regression was observed when assayed with mouse blood and BAL. As shown in Fig. 5b, when using F1-strips to analyze F1 or *Y. pestis* in mouse blood, the detection limits dropped to 100 ng and10^5^ CFU/ml, respectively. In BAL (Fig. 5c), the level of sensitivity for *Y. pestis* decreased to 10^4^ CFU/ml, and the sensitivity to F1 protein also been affected (data not shown).

**Table 3:** Evaluation of F1/*Y. pestis* in simulated samples. The human serum has the same detection limit as that in PCB (10^3^ CFU/ml and 4 ng/ml), while the limitation has decreased for mouse blood and BAL

Specimen types	PCB				Human serum				Mouse blood				Bronchoalveolar lavage			
	*Y. pestis* (CFU mL^− 1^)		F1 protein (ng mL^− 1^)		*Y. pestis* (CFU mL^− 1^)		F1 protein (ng mL^− 1^)		*Y. pestis* (CFU mL^− 1^)		F1 protein (ng mL^− 1^)		*Y. pestis* (CFU mL^− 1^)		F1 protein (ng mL^− 1^)	
	10^6^	**+**	10	**+**	10^6^	**+**	10	**+**	2 × 10^7^	**+**	1000	**+**	2 × 10^7^	**+**	1000	**+**
	10^5^	**+**	5	**+**	10^5^	**+**	5	**+**	2 × 10^6^	**+**	100	**+**	2 × 10^6^	**+**	100	**+**
	10^4^	**+**	4	**+**	10^4^	**+**	4	**+**	2 × 10^5^	**+**	10	**+**	2 × 10^5^	**+**	10	**+**
	10^3^	**+**	3	**±**	10^3^	**+**	3	**±**	2 × 10^4^	**±**	5	**±**	2 × 10^4^	**+**	5	**±**
	10^2^	**±**			10^2^	**±**							2 × 10^3^	**±**		

In addition, the Mab/Pab F1-strips were applied to field captured rodent specimens (the rodents were sacrificed with the method same as that of ICR mice). The results revealed that when these two types of strips were used for plague detection, same results were obtained. Although fleas were detected in some of these rodents (13/48), all rodents were F1 negative (data not shown. On the other hand, half of the samples were re-assayed by ELISA (blind test, data not shown), and the results were identical to the strip assay. These results implied that *Y. pestis* does not exist in selected areas; hence, the plague is a rare occurrence on Penghu Island.

## Discussion

This study describes a rapid detection of *Y. pestis* and recombinant F1 protein (the F1-antigen) in simulated clinical samples. For F1-protein purification, two of the most common techniques, size exclusion chromatography (SEC) and the anion exchange chromatography (AEC), were employed. In previous reports, Wong et al. [26] and Andrews et al. [43] used an SEC column to purify the F1 protein, and García-Otero et al. [27] showed good results in characterizing metals bound to marine dissolved organic matter using SEC followed the AEC. In this study, both columns were used to purify the F1-protein. We utilized the AEC in the second step to reduce lipopolysaccharides in the final purification product. However, neither product should be discarded when beginning with the AEC then the SEC, which means, the two products need to be pooled together for further purification. Nevertheless, the purity obtained by running two columns is higher than that of using only one column, though the recovery was ultimately reduced.

In this study, an immunosandwich antibody-antigen-antibody format was employed for immuno- chromatography assays. Two different mouse monoclonal anti-F1 antibodies (4B5–3 and 5F3–2) were available to act as either the capture antibody (bind onto colloidal gold particles) or the detection antibody (on strip membrane). The optimal antibody concentration was obtained by serial dilution testing, and this concentration was consistent for subsequent assays, and the results of the LFA should be recorded within 15 min. Our experience demonstrated that the chance of error increases after 15 min of incubation, because the mixture on the absorbent pad “migrates back” and further reacts with the antibodies on the membrane. This would ultimately enhance the signal and result in a pseudo-positive band.

The reason we used simulated samples rather than actual clinical samples was that in Taiwan, plague cases are almost extinct. Since BAL (or blood) has been widely used as a diagnostic tool of lung diseases, we instead used these simulated samples spiked with F1/*Y. pestis* diluents to mimic clinical assays using the in-house prepared F1 test strips. Because these undiluted simulated samples cannot flow smoothly for viscosity, the samples were therefore diluted to facilitate flow along the membrane. Figure 5 shows that when blood or lavage samples were spiked with various amounts of antigens, the detection sensitivity decreased to some extent (10^4^~ 10^5^ CFU/ml for *Y. pestis* or 10~ 100 ng/ml for F1 protein). The reason for this decline was not clear, but was probably because some combinations of blood cells/BAL fluids and F1/*Y. pestis* formed big particles that were unable to flow on the membrane. In addition, unknown component(s) that could interfere with the antigen-antibody reaction might have been present in blood cells/BAL. However, since the serum contained no blood cells or unknown components, hence the serum retained base-line sensitivity.

A previous study announced that LFA, based on purified F1 from *Y. pestis* EV76 strain and using the paired format of polyclonal and monoclonal antibodies, serum detection limit could up to 3.3 ng/ml [17]. Chanteau et al. [44] also described that when using LFA to diagnosis the plaque, the sensitivity of the assay was up to 0.5 ng/ml for clinical human samples [44]. On the other hand, when using a fiber optics biosensor with fluorescence-labeled antibodies, Cao et al. [23] also detected less than 5 ng/ml of F1-antigen [23]. In this study, we obtained similar results (4 ng/ml of F1-antigen) with in-house F1-strips. Although different lab set up or different test matrix could be used for the rapid assays, the LFA system needs to be tested with other ones’ side by side in order to provide its sensitivity and specificity advantage accurately. Thus we presume, the detection limit for the F1 protein depended on several aspects, such as the bacterial strain, the infected species, and the infection routes, among others. In other words, the detection sensitivity should be evaluated under same condition; it could be different when different species, different bacteria strains, or different routes were used.

In the cross-reactivity assay of this study, although various bacteria (1 × 10^5^ CFU/ml of each) were applied to the strips, only *Y*. *pestis* showed a positive result, indicating the high efficiency and specificity of the F1-strips. Based on the results, we assume that the F1-strips have sufficient sensitivity to detect *Y. pestis* in real situations.

For the rapid diagnosis of plague disease; LFA includes several benefits: it is a one-step assay requires less operating time, a user-friendly format, low chromatographic separation interference, relatively low manufacturing costs, and no requirement for special training or skilled technicians. These characteristics suggest that the strip assay is ideally suited for onsite testing by untrained personnel. However, it is noteworthy that some virulent *Y. pestis* strains might either produce low levels of the F1-antigen or lack the F1-antigen altogether. As a result, these strips cannot detect F1-negative *Y. pestis* strains; such capsular-negative strains have been isolated mostly from symptomatic plague patients. For this reason, it is of great importance to develop a robust non-F1-based detection system for *Y. pestis* F1-negative strains. Our goal is to develop a more specific method that is capable of detecting both F1-positive and F1-negative *Y. pestis* strains. We hope that this can be achieved in the near future.

## Conclusion

LFA represents a well-established and appropriate technology for the rapid diagnosis of plague disease. In this report, high levels of sensitivity and specificity of LFA indicated that F1 protein/*Y. pestis* could be directly detected. This report indicates that the strip assay is sufficiently sensitive to support the detection of plague disease in simulated clinical samples, and may be a good candidate to meet the epidemiological surveillance requirements during on-site and/or biological warfare.

## Abbreviations

CFU: Colony forming unit
ddH_2_O: Distillation-distillation H_2_O
ELISA: Enzyme linked immunosorbent assay
LFA: Lateral flow assay
MAb: Monoclonal antibody
NC membrane: Nitrocellulose membrane
PCB: Potassium carbonate buffer
